# A method for generating dynamic compression shear coupled stress loading on living cells

**DOI:** 10.3389/fbioe.2022.1002661

**Published:** 2022-09-21

**Authors:** Dasen Xu, Nu Zhang, Sijie Wang, Pan Zhang, Yulong Li, Hui Yang

**Affiliations:** ^1^ School of Aeronautics, Northwestern Polytechnical University, Xi’an, China; ^2^ Center of Special Environmental Biomechanics; Biomedical Engineering, Northwestern Polytechnical University, Xi’an, China; ^3^ School of Life Science, Northwestern Polytechnical University, Xi’an, China; ^4^ School of Civil Aviation, Northwestern Polytechnical University, Xi’an, China; ^5^ Joint International Research Laboratory of Impact Dynamic and Its Engineering Application, Xi’an, China

**Keywords:** dynamic compression-shear coupling stress, Device development, cell mechanics, CFD model, weak shock wave

## Abstract

Changes in the mechanical properties of single cells are related to the physiological state and fate of cells. The construction of cell constitutive equations is essential for understanding the material characteristics of single cells. With the help of atomic force microscopy, bio-image processing algorithms, and other technologies, research investigating the mechanical properties of cells during static/quasi-static processes has developed rapidly. A series of equivalent models, such as viscoelastic models, have been proposed to describe the constitutive behaviors of cells. The stress-strain relations under dynamic processes are essential to completing the constitutive equations of living cells. To explore the dynamic mechanical properties of cells, we propose a novel method to generate a controllable dynamical compression shear coupling stress on living cells. A CFD model was established to visualize this method and display the theories, as well as assess the scope of the application. As the requirements or limitations are met, researchers can adjust the details of this model according to their lab environment or experimental demands. This micro-flow channel-based method is a new tool for approaching the dynamic mechanical properties of cells.

## Introduction

During the processes of cell development, differentiation, physiology, and disease, cells receive not only chemical signals but also mechanical signals from the extracellular matrix and surrounding environment (Hamed, 2020). Mechanical forces are experienced by cells and may be interpreted as a signal to induce phenotypic and functional responses or pathways, such as gene expression cascades, protein synthesis, proliferation, and movement; these responses can temporarily or even permanently change the cellular state (Desprat et al., 2005; Patterson et al., 2019). Moreover, the mechanobiological responses of biological cells had been extensively studied also, e.g., the responses of mesenchymal stem cells and chondrocytes to mechanical stimuli (Zhang et al., 2008; Zhang et al., 2012; Ganadhiepan et al., 2019). From a mechanical perspective, cells present a special material that is far more complex than ordinary materials, such as metal and glass. It is worth noting that the mechanical properties of cells remain unstable in most pathological processes, such as metastasis, asthma, sickle cell anemia, and apoptosis (Alberts et al., 2002; Desprat et al., 2005; Hao et al., 2020). Thus, understanding the mechanical behaviors of cells can provide a useful perspective on describing disease progression and revealing the fundamental mechanisms of the working of biomaterials (Bao and Suresh, 2003; Moeendarbary and Harris, 2014).

In exploring the mechanical behaviors of cells and establishing stress-strain relationships in them, it is a challenge to properly apply a controllable force on the tissue/monolayer/cell and capture its real-time strain at a single cell scale (i.e., $10^{-5}$ m) (Patterson, 2020). In this regard, various reasonable assumptions have been proposed, which present a research-scale perspective on mechanical methods, including the mechanical and biological methods (Moeendarbary and Harris, 2014; Hao et al., 2020).

In conjunction with atomic force microscopy (AFM), the bio-image processing algorithm (Dudani et al., 2013), micropipette aspiration (MA) (Evans and Yeung, 1989), and microfluidic platforms (Urbanska et al., 2020) are the most common and effective mechanical tools to apply compression/tensile or shear stress on cells. In addition, to improve accuracy and collect more information, some modified techniques and methods have been designed, such as magnetic twisting cytometry (MTC) (Trepat et al., 2004) and uniaxial stretching rheometer (USR) (Desprat et al., 2005). Through these static or quasi-static mechanical experiments, it is believed that the mechanical behavior of cells is likely to resemble that of viscoelastic material (Katti et al., 2019). However, experiments exploring the stress-strain relationship of isolated living cells did not reach dynamic conditions or higher strain rates ( $\dot{\epsilon }>10^{-1}$ s^−1^). Commonly, the dynamic loading process of materials, including living cells, differs significantly from the static or quasi-static situations. For instance, a quasi-static deformation situation comprises a sequence of equilibrium states, where the well-known equations describing the mechanics of materials work (i.e., ∑F=0 ; ∑M=0 ). On the contrary, the dynamic loading process can be treated as a stress wave traveling through the body at an acoustic speed (Meyers, 1994). When an external deformation is imparted at a very high rate, it induces stress on one portion of the body, while other portions remain unaffected. As cells can sense mechanical behaviors, they react rapidly to adapt to them (Pelham and Wang, 1997). Cells subjected to dynamic loading processes exhibit distinct mechanical behaviors rather than viscoelastic material. Moreover, the stress-strain relations under dynamic processes are an important part of the single-cells constitutive equations. Therefore, developing methods to apply dynamic stress on cells will significantly promote the understanding of the mechanical behaviors of cells under dynamic conditions (Bao and Suresh, 2003).

In the present work, we propose a novel method to apply combined dynamic compression-shear loading on isolated living cells under normal conditions; in addition, we extend the range of stiffness tensor of single biological cell piecewise function to higher strain rates ( $\dot{\epsilon }>10^{0}$ s^−1^). The basis of this new method is the theory of transient flow, or in detail, the theory of the weak shock wave (where “weak” signifies that the thermal energy generated by impact compression is smaller compared to the total internal energy of the fluid (Smith, 1973)) propagation in a viscous fluid, which would suddenly induce both the compression and shear stresses in the boundary layer. In addition, the water hammer theory asserts that we can repeatedly apply disturbance with the amplitude of the weak shock wave, which is precisely controllable by changing the speed of the projectile. Briefly, this method includes two parts: the stress loading part accelerates a projectile to impact a fluid-fulfilled microchannel seeded with living cells on the bottom, while the strain acquisition part is equipped with a high-speed camera to assist with an image processing algorithm. Once the assumptions and requirements are fulfilled, the details of the design remain readily changeable. The proposed methods can be used to explore the stress-strain relations under dynamic processes and clarify the constitutive behavior of single cells to dynamic loadings.

## Methods

**FIGURE 1:**
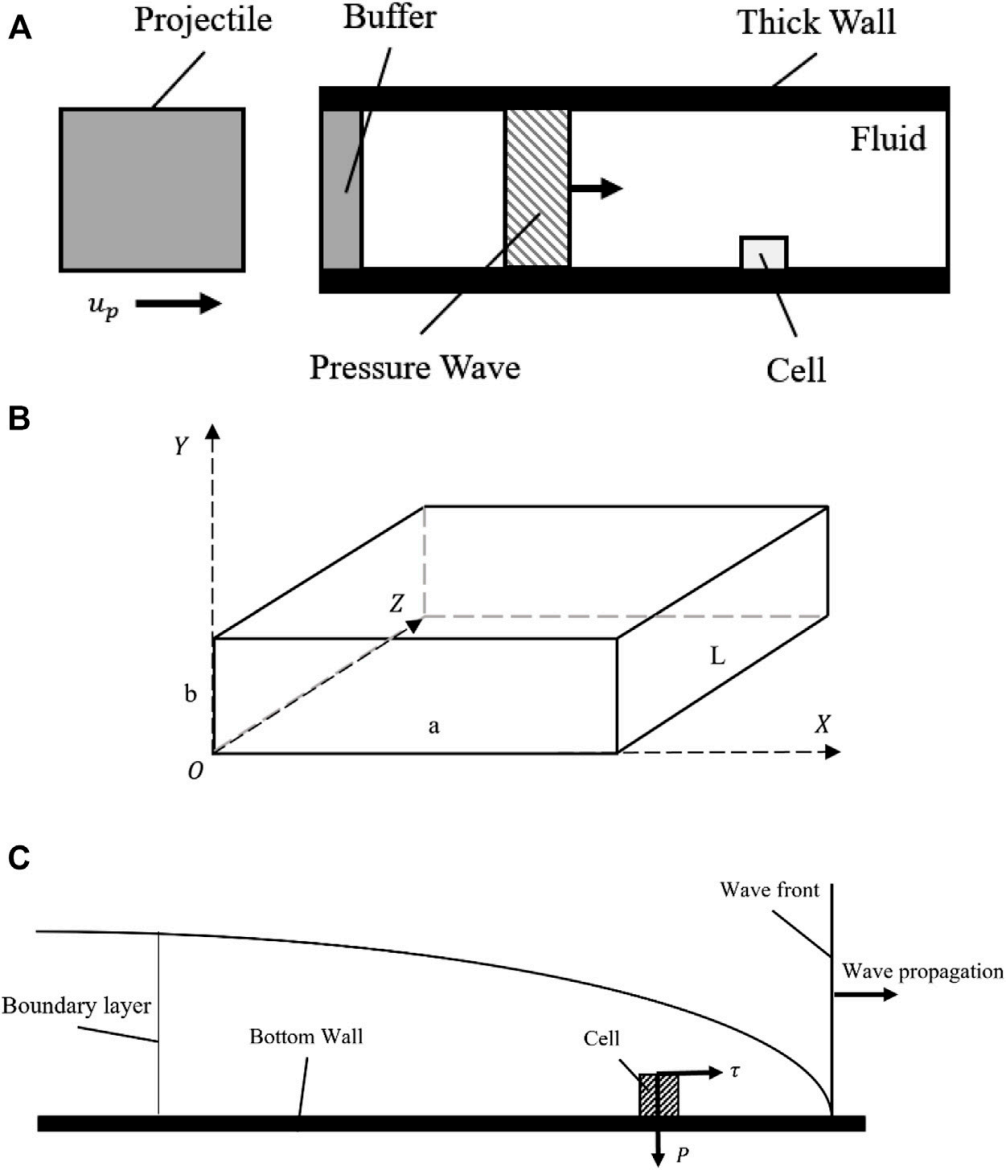
A schematic diagram showing the basics of the proposed model. **(A)** The schematic diagram of the geometry of the model; **(B)** The sketch of the rectangular channel; **(C)** The stress analysis of cells during wave propagation.

To illustrate the dynamic and coupled compression-shear loading method, a simple schematic diagram of the model is presented in Figures 1A,B. The model exhibits a gas gun, projectile, and a microfluidic chip with a rectangular conduit channel, where cells can be seeded on the bottom wall. In this mode, a projectile was accelerated to an initial velocity ( $u_{p}$ ) to impact the buffer; a pressure surge was then generated, which traveled through the fluid matter. As this stress wave propagated at the acoustic velocity, the cells cultured on the bottom wall experienced the compression stress by the stress wave directly, as well as shear stress due to the viscosity of the fluid (Figure 1C).

### Navier-Stokes equations

To allow the pressure waves to propagate, the fluid used in this experiment was compressible with constant viscosity. Therefore, the equations of continuity and momentum (Navier-Stokes equations) used to describe the motion of the fluid are written as:

$$\begin{array}{l} \frac{\partial \rho }{\partial t}+\nabla \cdot \left(\rho u\right)=0 \end{array}$$ (1)

$$\begin{array}{l} \frac{\partial \left(\rho u\right)}{\partial t}+\nabla \cdot \left(\rho u\cdot u\right)=-\nabla P+\mu \nabla^{2}u+\frac{\mu }{3}\nabla \left(\nabla \cdot u\right) \end{array}$$ (2)

Where ρ is the fluid density; μ is the dynamic viscosity; u is the fluid velocity; ″∇″ is the gradient operator; ″∇⋅″ is the divergence operator; $\prime \prime \nabla^{2}\prime \prime$ is the Laplace operator which means ″∇⋅∇″ ; t is time, and P is the total pressure in the fluid. For isotropic and homogeneous newton fluid, the stress tensor can be expressed as:

$$\begin{array}{l} \sigma_{ii}=-P+2\mu \frac{\partial u_{i}}{\partial x_{i}}-\frac{2}{3}\mu \nabla \cdot u \end{array}$$ (3)

$$\begin{array}{l} \tau_{ij}=\mu \left(\frac{\partial u_{i}}{\partial x_{j}}+\frac{\partial u_{j}}{\partial x_{i}}\right) \end{array}$$ (4)

Where $u_{i}$ is the fluid velocity, and $x_{i}$ is the spatial coordinate ( i=1, 2, 3 ). $\sigma_{ii}$ is the pressure components of stress tensor, and $\tau_{ij}$ is the shear components of stress tensor. In the channel flow with non-slip wall condition, the wall shear stress $\left(\tau_{i}^{w}\right)$ could be calculated using:

$$\begin{array}{l} \tau_{i}^{w}=\mu \left(\frac{\partial u_{i}}{\partial n_{w}}\right) \end{array}$$ (5)

Where $n_{w}$ is the distance normal to the wall.

### Wave propagation celerity in a rectangular conduit channel

A general solution for the celerity of pressure wave propagating in the fluid has been derived by Hutarew and can be applied for a conduit of any cross-section (Hutarew, 1973). The form is:

$$\begin{array}{l} c_{f}=1/\left(\rho \left(\frac{1}{K}+\frac{1}{A}\frac{\delta A}{\delta p}\right)\right) \end{array}$$ (6)

Where *K* is the fluid bulk modulus, A is the cross-section area of the channel, and $c_{f}$ is the wave propagation celerity. δ is a small change sign. Eq. 6 shows the effect of fluid-structure interaction on the propagation speed of the pressure surge. This means that despite the fluid in the Computational Fluid Dynamics (CFD) model being considered compressible, the deformation of channel walls should be taken into account. However, in this study, a rigid-walled boundary condition was preferred, which could make the measurement and calculation of the cell strain field much more concise. Hence, a corrected fluid bulk modulus $\left(K_{\mathrm{mod}}\right)$ was proposed to incorporate structural behavior in he CFD model. For the propagation of pressure surge in liquids traveling through thick-walled pipes and ducts of rectangular cross-section, the theoretical wave celerity equation was given by Thorley (Thorley and Guymer, 1976):

$$\begin{array}{l} c_{f}=1/\left(\rho \left(\frac{1}{K}+\frac{\Phi \left(a,b\right)}{abEe^{2}}\right)\right) \end{array}$$ (7)

$$\Phi \left(a,b\right)=\frac{a^{3}+b^{3}}{2\left(a+b\right)}\left(\frac{a^{3}}{6}+\frac{a^{2}b}{2}-\frac{b^{3}}{3}\right)-\frac{a^{5}}{20}\begin{array}{l} -\frac{a^{2}a^{3}}{4}+\frac{b^{5}}{5}+\frac{Ee^{2}}{4G}\left(a^{3}+b^{3}\right)+\frac{abe^{2}}{2}\left(a+b\right) \end{array}$$ (8)

Where *a* and *b* are the lengths of the long and short sides of the rectangular cross-section; *e* is the wall thickness; E and *G* are the elastic and shear moduli of the wall material, respectively. Φ(a,b) was solved using Eq. 8. According to the equation defining wave propagation velocity, the corrected fluid bulk modulus $K_{\mathrm{mod}}$ equation is:

$$\begin{array}{l} c_{f}={\left(\frac{K_{\mathrm{mod}}}{\rho_{0}}\right)}^{\frac{1}{2}} \end{array}$$ (9)

Nevertheless, the properties of solids involved played an important role in wave propagation as per the wave celerity equation. An evaluation of the fluid-solid coupling effect was needed to examine the limitations when the motion of the channel was rigid. A dimensional parameter “ β ”, named “fluid loading”, was proposed by Pinnington to evaluate the fluid-solid coupling; it corresponded to the Korteweg wave speed equation (Korteweg, 1878; Pinnington, 1997; Shepherd and Inaba, 2009).

$$\begin{array}{l} \beta =\left(c_{0}/c_{f}\right) \end{array}$$ (10)

$$\begin{array}{l} \beta =\left(\frac{c_{f}^{2}}{c_{s}^{2}}\right)\left(\frac{\rho_{f}}{\rho_{s}}\right)\left(\frac{2R}{e}\right) \end{array}$$ (11)

Where $c_{0}$ is the acoustic speed in fluid; R is the mean radius of the channel; the subscript, “ s ”, denotes a structure, and the subscript, “ f ”, means fluid. Moreover, according to the limiting cases of fluid-structure interaction (FSI) discussed by Shepherd and Inaba, the case, where ≪1 , indicated that the channel could be regarded as rigid (Shepherd and Inaba, 2009). Substituting the wave celerity and acoustic speed in Eq. 10 to get the parameter β helped us design the details of the channel.

### Pressure wave generation by projectile impact

To describe the fluid suitable here with equations, the Tait and Tammann equations of state, which apply to a wide range of liquids, were used (Dymond and Malhotra, 1988; Hoang et al., 2015). The Tait equation can be written as a function of pressure (p) and density (ρ)

$$\begin{array}{l} p=B{\left(\frac{\rho }{\rho_{0}}\right)}^{\gamma \;}-B \end{array}$$ (12)

Where B is a weak function of entropy, which is usually treated as a pressure constant of $3.35\times 10^{8}$ Pa. γ is the adiabatic exponent that equals 7.15 (Nagayama et al., 2002). The Tait equation was rewritten as a partial differential equation form.

$$\ln\left(p+B\right)=\ln\left(B\right)+\gamma \ln\left(\frac{\rho }{\rho_{0}}\right)$$

$$\begin{array}{l} \frac{\partial p}{\partial \rho }=\frac{\gamma \left(p+B\right)}{\rho } \end{array}$$ (13)

As the whole process was assumed to be isentropic, the adiabatic exponent γ was:

$$\begin{array}{l} \gamma =-\frac{\gamma }{p}{\frac{dp}{d\gamma }|}_{s} \end{array}$$ (14)

Where γ is the volume Upon substituting volume γ by density ρ, we obtained:

$$\begin{array}{l} \frac{p}{p_{0}}={\left(\frac{\rho }{\rho_{0}}\right)}^{-\gamma } \end{array}$$ (15)

The definition of acoustic velocity is:

$$\begin{array}{l} c^{2}=\left(\frac{\partial p}{\partial \rho }\right) \end{array}$$ (16)

Upon integration of Eq. 16, a function of pressure and wave speed describing the fluid density was obtained, which could be used to correct the density in CFD, as the fluid was considered compressible.

$$\begin{array}{l} \rho =\rho_{0}+c^{-2}p \end{array}$$ (17)

The acoustic velocity is derived from Eq. 15.

$$\begin{array}{l} {\left(\frac{\partial p}{\partial \rho }\right)}_{s}=\gamma \left(\frac{p_{0}}{\rho_{0}}\right){\left(\frac{\rho }{\rho_{0}}\right)}^{\gamma -1\;}=c^{2} \end{array}$$ (18)

As the compressibility of the fluid was limited, an approximation of the acoustic velocity under normal conditions could be written as:

$$\begin{array}{l} \lim_{\rho \to \rho_{0}}{\left(\frac{\partial p}{\partial \rho }\right)}_{s}=\frac{\gamma p_{0}}{\rho_{0}}=c_{0}^{2} \end{array}$$ (19)

The local acoustic velocity could be written as:

$$\begin{array}{l} c={\left(\frac{\partial p}{\partial \rho }\right)}^{\frac{1}{2}}=c_{0}{\left(\frac{p}{p_{0}}\right)}^{\frac{\gamma -1}{2\gamma }\;}=c_{0}{\left(\frac{\rho }{\rho_{0}}\right)}^{\frac{\gamma -1}{2\gamma }} \end{array}$$ (20)

**FIGURE 2:**
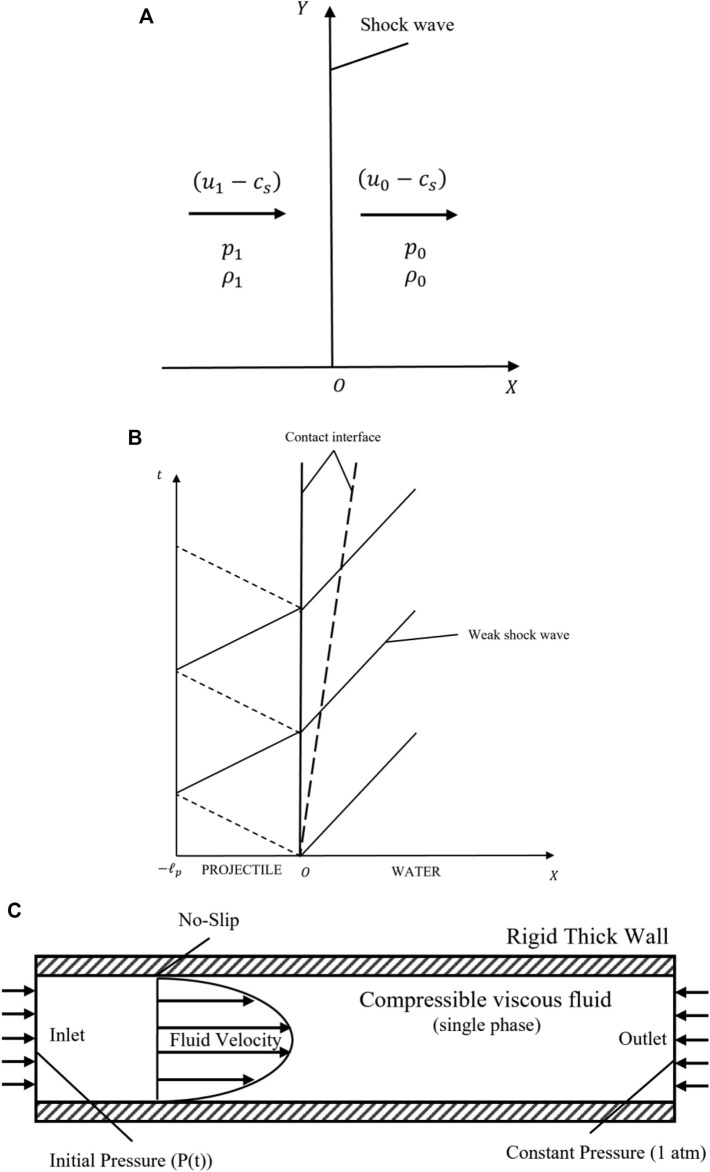
The isolation of boundary conditions and analysis of the model. **(A)** The weak shock wave jumps conditions under a coordinate fixed with the shock wave front; **(B)** The weak shock wave generated by projectile impact analysis; **(C)** A schematic diagram illustrating the micro-channel boundary conditions in CFD modeling.

Figure 2A displays the shock wave jumps conditions with a coordinate fixed at the shock wave front. The equations of conservation (mass; momentum; energy) could be written as:

$$\begin{array}{l} \rho_{0}\left(u_{0}-c_{s}\right)=\rho_{1}\left(u_{1}-c_{s}\right) \end{array}$$ (21)

$$\begin{array}{l} \rho_{0}\left(u_{0}-c_{s}\right)u_{0}dt=\rho_{1}\left(u_{1}-c_{s}\right)u_{1}dt \end{array}$$ (22)

$$\begin{array}{l} \frac{1}{2}u_{0}^{2}+\frac{\gamma }{\gamma -1}\frac{p_{0}}{\rho_{0}}=\frac{1}{2}u_{1}^{2}+\frac{\gamma }{\gamma -1}\frac{p_{1}}{2} \end{array}$$ (23)

These equations are usually known as the Rankine-Hugoniot equations (Smith, 1973). Here, we neglected the thickness of the shock front, and thus, the particle velocity, u and shock velocity, $c_{s}$ behind the shock front could be solved. For very weak shock waves, the jump process could be treated as an isentropic process, yielding the following equation:

$$\begin{array}{l} c_{s}={\left(\left(\frac{\rho }{\rho_{0}}\right)\;\left(\frac{p-p_{0}}{\rho -\rho_{0}}\right)\right)}^{\frac{1}{2}} \end{array}$$ (24)

$$\begin{array}{l} u=\frac{\left(\rho -\rho_{0}\right)}{\rho }\;c_{s} \end{array}$$ (25)

$$\begin{array}{l} \frac{\rho_{1}}{\rho_{0}}=\frac{\left(\gamma +1\right)p_{1}+\left(\gamma -1\right)p_{0}}{\left(\gamma +1\right)p_{0}+\left(\gamma -1\right)p_{1}}=\frac{u_{0}}{u_{1}} \end{array}$$ (26)

The particle velocity could then be rewritten with Eq. 19.

$$\begin{array}{l} u=\frac{p-p_{0}}{\rho_{0}c_{0}}=\frac{c_{0}}{\gamma p_{0}}\;\left(p-p_{0}\right) \end{array}$$ (27)

or

$$\begin{array}{l} \frac{p}{p_{0}}=1+\frac{\gamma u}{c_{0}} \end{array}$$ (28)

Substituting by Eq. 20, gave:

$$\begin{array}{l} c_{s}=c_{0}{\left(1+\frac{\gamma u}{c_{0}}\right)}^{\frac{\gamma -1}{2\gamma }} \end{array}$$ (29)

The Taylor expanded form of Eq. 29 thus obtained is:

$$c_{s}=c_{0}\left(1+\frac{\gamma -1}{2c_{0}}u+O\left(u^{2}\right)+\ldots \right)≅c_{0}\left(1+\frac{\gamma -1}{2c_{0}}u\right)\begin{array}{l} ≅c_{o}+\left(\frac{\gamma -1\;}{2}\right)u \end{array}$$ (30)

As the relationship between acoustic velocity and particle velocity was obtained, the differential form was:

$$\begin{array}{l} du_{p}=du=\frac{2}{\gamma -1}\;dc_{S} \end{array}$$ (31)

As no cavitation or fluid column separation occurred and no cross-section changes were recorded along the channel, the Joukowsky equation was a perfect approximation to predict the maximum pressure in a water-hammer impact situation (Joukowsky, 1900; Walters and Leishear, 2018). This impact occurs at (x,t)=(0,0) , and an equation, p(t) , describes the dynamic pressure of the fluid at the interface of the fluid and projectile. This initial impact would create a weak shock wave with an amplitude that can be determined by the pressure-velocity matching method (Meyers, 1994; Deshpande et al., 2006; Shepherd and Inaba, 2009)

$$\begin{array}{l} \Delta p=p\left(0\right)=\frac{{\left(\rho c\right)}_{f}{\left(\rho c\right)}_{p}}{{\left(\rho c\right)}_{f}+{\left(\rho c\right)}_{p}}u_{p} \end{array}$$ (32)

Where ${\left(\rho c\right)}_{f}$ is the fluid impedance; ${\left(\rho c\right)}_{p}$ is the projectile impedance. The impedance of a projectile is much higher than that of the fluid in most cases, and Eq. 12 can reduce it to an approximated expression:

$$\begin{array}{l} p\left(0\right)\approx {\left(\rho c\right)}_{f}u_{p} \end{array}$$ (33)

After the conditions of the fluid were solved, the impact condition obtained is shown in Figure 2B. The shock wave generation due to the projectile impact was a discrete process. In this study, since the buffer was assumed to be as thin as possible, it could be neglected given that it had the same impendence as the projectile. This model could be simplified to a projectile impacting the fluid column directly. Analysis of the projectile motion was performed according to Newton’s second law.

$$\begin{array}{l} p_{i}A_{p}=m_{p}\frac{du_{p}}{dt} \end{array}$$ (34)

Where $p_{i}$ is the pressure by every discrete impact; $u_{p}$ is the interface velocity of projectile and fluid; $A_{p}$ is the cross area of the projectile, and $m_{p}$ is the mass of the projectile. Substituting Eq. 34 with Eq. 31, the result was:

$$\begin{array}{l} p=\frac{2}{\gamma -1}\frac{m_{p}}{A_{p}}\frac{dc_{s}}{dt} \end{array}$$ (35)

Meanwhile, Eq. 12 was rewritten in terms of local acoustic velocity $c_{s}$ to obtain the following:

$$\begin{array}{l} p=B{\left(\frac{\rho_{0}c^{2}}{nB}\right)}^{\frac{\gamma }{\gamma -1}}-B \end{array}$$ (36)

The partial differential form of Eq. 33 is:

$$\begin{array}{l} \frac{\partial p}{\partial c}=\frac{2\gamma }{\gamma -1}\frac{P+B}{c} \end{array}$$ (37)

In our model, the pressure was too small as compared with the pressure constant, which is $3.35\times 10^{8}$ Pa (Nagayama et al., 2002). A linear approximation treatment (Lennon, 1994) was used with normal condition parameters $\left(p_{0},\;c_{0}\right)$ to calculate Eq. 34, which could be rewritten as:

$$\begin{array}{l} {\left(\frac{\partial p}{\partial c}\right)}_{s}=\frac{2\gamma }{\gamma -1}\frac{P_{0}+B}{c_{0}} \end{array}$$ (38)

The normal conditions here were $p_{0}=101325\;Pa$ , $\rho_{0}=999.8$ kg/m^3^, and $c_{0}=1439$ m/s. Eq. 33 was then rewritten as follows:

$$\begin{array}{l} p=\frac{2}{\gamma -1}\frac{m_{p}}{A_{p}}\frac{dc_{s}}{dt}=\frac{2}{\gamma -1}\frac{m_{p}}{A_{p}}{\left(\frac{\partial c}{\partial p}\right)}_{s}\frac{dp}{dt} \end{array}$$ (39)

Upon integration of Eq. 36, a simplified function of pressure was gained.

$$\begin{array}{l} p\left(t\right)=p\left(0\right)\exp\left(-\lambda t\right) \end{array}$$ (40)

Where the time constant was as follows:

$$\begin{array}{l} \lambda =\frac{\gamma -1}{2}\frac{A_{p}}{m_{p}}{\left(\frac{\partial p}{\partial c}\right)}_{s} \end{array}$$ (41)

### Boundary layer induced by a very weak shock wave

Once a weak shock wave enters a static fluid boundary through the wall, a boundary layer begins to appear at the interaction point of the weak shock wave and the wall (Ackroyd, 1967; Davies and Bernstein, 1969). A study of the laminar compressible boundary layer induced by this weak shock wave was solved by H. Mirels’ in 1955, who provided theoretical evidence to prove that the weak shock waves generated by projectile impact indeed induce a boundary layer (Mirels, 1955).

Considering a possible turbulent boundary layer, R. E Melnik and B. Grossman developed an asymptotic theory within the limit of weak shock waves (Melnik and Grossman, 1974). Their three-layer description of the boundary layer was a natural extension of the asymptotic theories of Mellor (Mellor, 1972), Yajnik (Yajnik, 1970), Bush and Fendell (Bush and Fendell, 1972; Bush and Fendell, 1973) for incompressible boundary layers, as well as the theory by Afzal (Afzal, 1973) for compressible non-interacting turbulent boundary layers. FLUENT is a reliable and acceptable CFD software employed for relevant numerical simulations. To simulate our model, the k-ε model was utilized to analyze turbulent flow (Versteeg and Malalasekera, 2007; Nikpour et al., 2014).

### Geometry definition

In the present work, the geometry definition consisted of a volume occupied by the flow with a specified shape of the physical boundaries. Here are several important criteria to limit the size of the channel, although, in the beginning, we planned to put the ‘lab’ into just one small chip to make this as convenient as possible. A fundamental restriction applied was the continuous flow in the fluid domain to ensure that the theories being considered were effective. A dimensionless parameter, the Knudsen number, was used to describe this problem (Guo et al., 2013); this number is defined as

$$\begin{array}{l} K_{n}=ƛ/\ell_{char} \end{array}$$ (42)

Where ƛ is the mean free path of the particle, and $\ell_{char}$ is the characteristic length scale. The fluid domain was described as a continuum and solved by Navier-Stokes (N-S) equations with no-slip boundary conditions, therefore, the $K_{n}$ was generally considered to be less than 0.001 (Ben-Dor et al., 2000; Kandlikar et al., 2005; Rapp, 2016).

According to the requirements above, we established a simple CFD model to describe our method. The Cartesian coordinates system was created as shown in Figure 1B, and a 3×3×60 mm rectangular conduit channel was drawn in ANSYS. The details of size were kept adjustable to adapt to various laboratory environments for other researchers. An example size is proposed here to describe dynamic stress loading methods.

### Mesh generation and solver settings

Transient simulation is strongly dependent on the quality of the mesh. For most water-hammer models to accurately capture the near-wall velocity, the mesh density near the wall should be concentrated. During the cross-section meshing process, the boundary layer neighboring the wall was divided into 20 layers with the first layer having a thickness of $1\times 10^{-6}$ m (1.1 growth rate); this design was based on the mesh independence analysis of 3D pressurized pipe flow with CFD modeling previously described by Martins et al. (Martins et al., 2018). Given the axial direction, a sweep method was applied. The Courant-Friedrichs-Lewy (CFL) criterion was used to maintain stability during the movement of the acoustic wave across the discrete elements in CFD, which determined the element size in the axial direction. The non-dimensional Courant number was calculated using the following equation:

$$\begin{array}{l} C_{courant}=c_{f}\frac{\Delta t}{\Delta z} \end{array}$$ (43)

Where Δz is the element size in the axial direction, and Δt is the calculation time step. There were two main considerations for the determination of $C_{courant}$ : one was to capture the wave velocity in the fluid; and the other was to calculate the stress on an arbitrary cell at the channel wall. The $C_{courant}$ was ideally expected to be ≤ 1. Moreover, to maintain the meshing quality, i.e., to keep the aspect ratio of all mesh elements < $10^{3}$ , the axial element size was Δz=0.1 mm with a time step of $t=0.7\times 10^{-7}$ s. The total time was assumed to be $4.2\times 10^{-5}$ s, i.e., 600 steps in total, to ensure that the entire compression wave could travel completely from inlet to outlet. The total mesh count was 1.98 million elements (Martins et al., 2014; Mandair, 2020).

In the CFD solver setup, boundary settings comprise the physical description of the fluid flow as shown in Figure 2C. The pressure applied on the inlet boundary has been described as a function of time, (t) in Eq. 40, which simulated the impact process. The outlet was operated under normal pressure ( $p_{0}=10135$ Pa), which could be achieved easily by collecting an extra tank filled with fluid, and the wall was set at a no-slip condition. In addition, there were two settings (heights) for the roughness of the bottom wall: $1\times 10^{-5}$ (average height of cells) and 0 m, which helped estimate the effect of cells on the fluid flow.

ANSYS Fluent^®^ (2019R3) was utilized to obtain all the simulation results presented in this paper. In FLUENT, the numerical tech is a finite volume method (FVM). The whole process is transient. As the fluid is considered to be viscous, compressible, isothermal (no heat transfer), isotropic, and single-phase (no cavitation), the semi-implicit method for pressure-linked equations (SIMPLE) can be used as a flow solver. Convergence was defined to be $1\times 10^{-6}$ due to the flow characteristics (Martins et al., 2016).

## Results

### Sample numerical results

**FIGURE 3:**
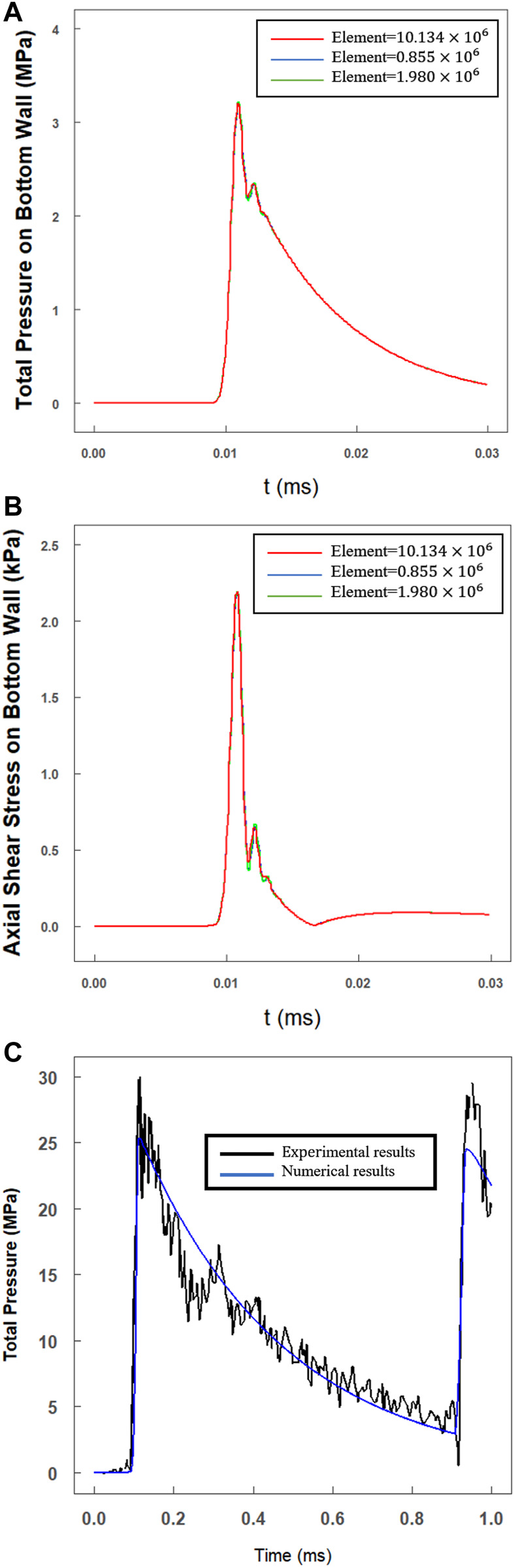
Mesh independence analysis and sample comparison results. The total mesh count for testing the independence were about $0.855\times 10^{6}$ (blue line), $1.98\times 10^{6}$ (green line), $10.134\times 10^{6}$ (red line) elements respectively. **(A)** The variations of total pressure on bottom wall at the monitoring point ( x=0.25 X ); **(B)** The variations of axial shear stress on bottom wall at the monitoring point ( x=0.25 X ); **(C)** A pressure waveform comparison of experimental results of example and numerical results (black line: experimental results; blue line: numerical results).

A mesh independence analyses were performed using different element size (the original element size was proposed in Section 2.6) by the simulation of pressure and shear stress on bottom wall. The total mesh count for testing the independence were about $0.855\times 10^{6}$ (blue line), $1.98\times 10^{6}$ (green line), $10.134\times 10^{6}$ (red line) elements respectively. The test results were shown in Figures 3A,B which assure a mesh independence of the simulation results in this paper.

We choose a classical water hammer experiment (Inaba and Shepherd, 2010) to assess the modeling method in our work. In this example (shot 62), researchers accelerated a steel projectile ( =0.67 kg, $v_{impact}=18.5$ m/s) to impact a specimen tube ( $D_{innerdiameter}=38.1$ mm, $h_{thick\;wall}=12.74$ mm), and the experimental pressure was recorded by strain gauges and a pressure transducer.

In Figure 3C, we compared the experimental results (black line; shot 62, gauge g1) with the numerical results (blue line) calculated with the modeling method in Section 2. The maximum pressure of 25.03 MPa was computed which showed a good match with the peak pressure of 27.20 MPa. Furthermore, the whole trend of the pressure waveform showed a good agreement with the experimental results. For instance, due to the outlet of the specimen tube being closed, a reflected wave could be observed.

### Visualization of pressure wave propagation

**FIGURE 4:**
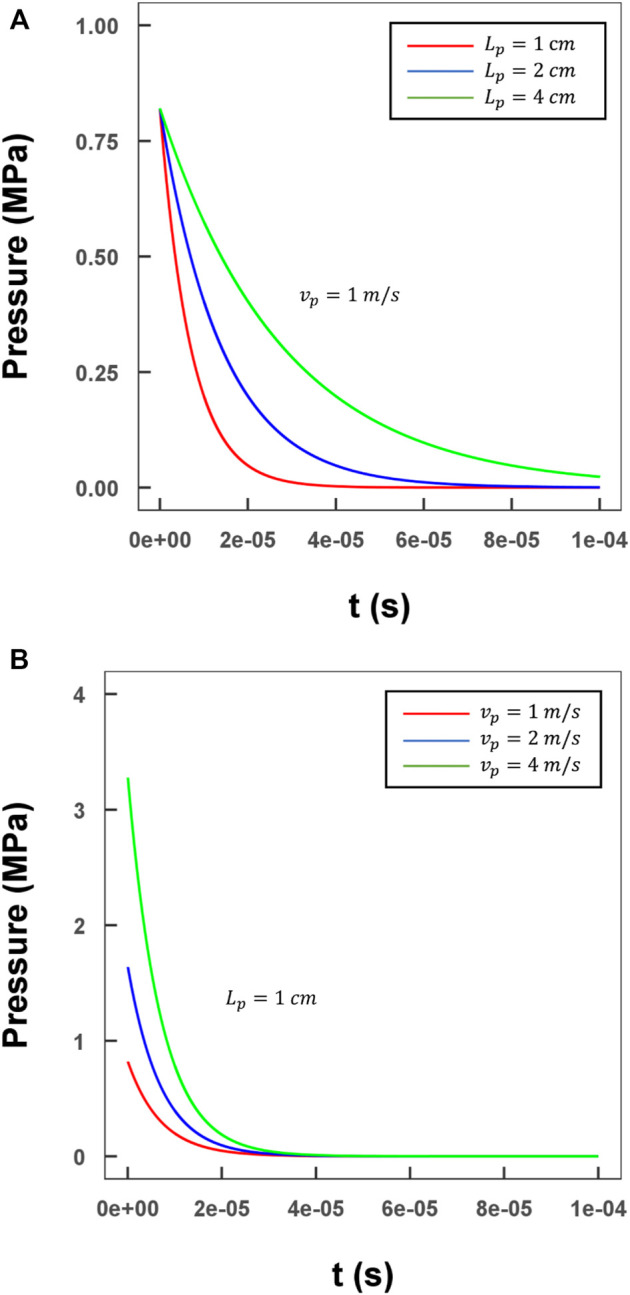
Evaluation of the relationship between various lengths/velocities of the projectile and the input pressure profile. **(A)** Three different lengths of projectile with a 1 m/s velocity (green line: 4 cm; blue line: 2 cm; red line: 1 cm); **(B)** Three different lengths of projectile with a 1 cm length (green line: 4 m/s; blue line: 2 m/s; red line: 1 m/s).

To visualize and analyze the wave propagation process in detail, as well as the profile of the coupled compression and shear stresses in our method, a representative case with a water-filled channel and a projectile made of polymethyl methacrylate (PMMA) was designed; a practical size was determined as described in Section 2.5. The correlation between the projectile parameters (velocity and length) and the input pressure was assessed, and the results are shown in Figure 4. The maximum value and the duration of the pressure input were adjustable by changing the length and initial velocity of the projectile. This detailed relationship was derived in Section 2.3 (Joukowsky, 1900; Versteeg and Malalasekera, 2007).

**FIGURE 5:**
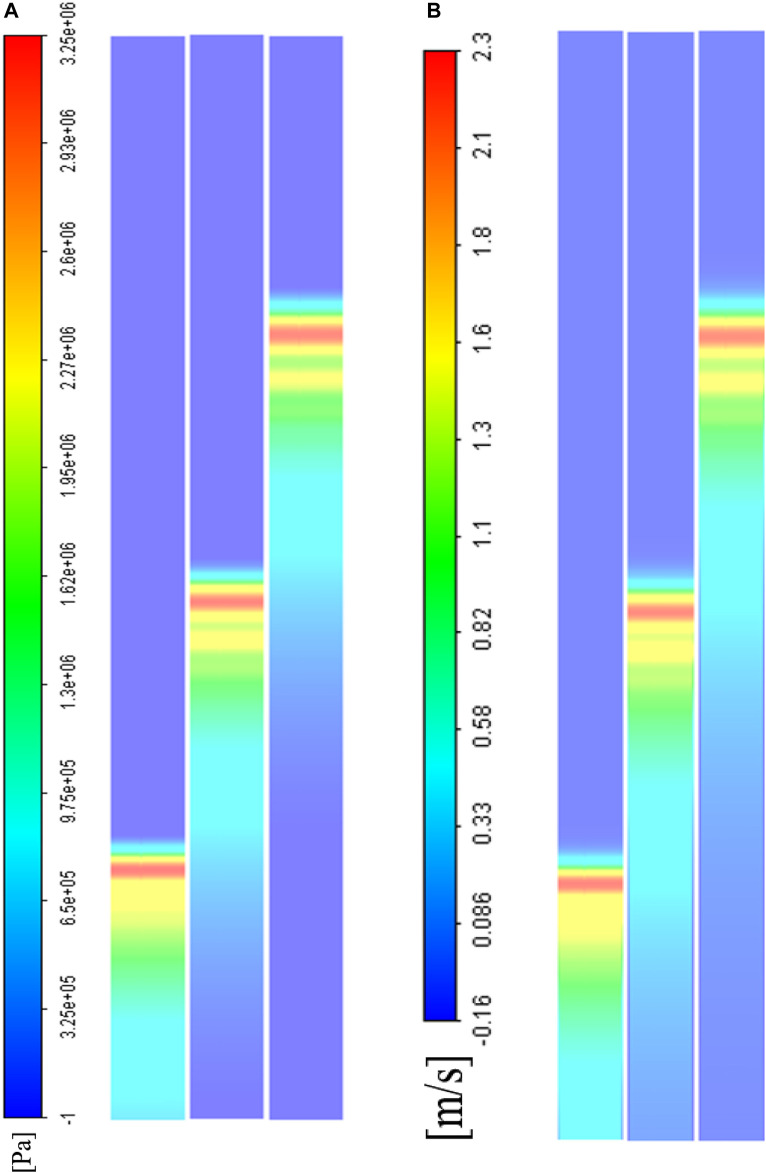
A CFD visualization of weak shock wave propagation in the middle axial cross-section at different time steps ( $t_{1}=0.25\;T,\;t_{2}=0.50\;T,\;t_{3}=0.75\;T$ ). **(A)** The total pressure field; **(B)** The axial velocity field.

**FIGURE 6:**
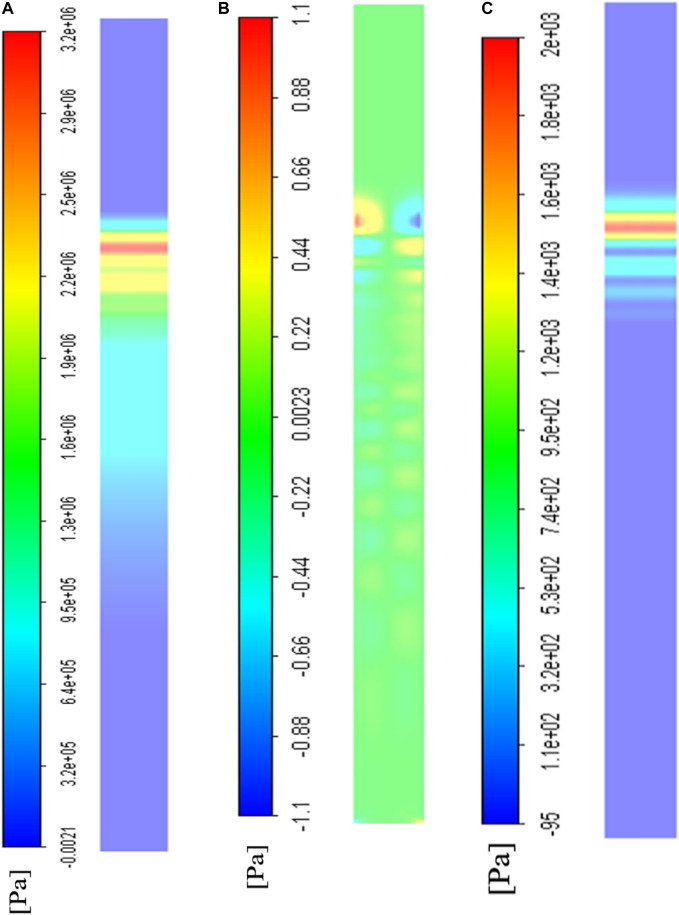
The CFD visualization of stress distribution on the bottom wall where cells were cultured at t=0.75 T time step. **(A)** The total compression stress distribution; **(B)** The transverse shear stress distribution; **(C)** The axial shear stress distribution.

As the inlet boundary condition, maximum pressure of 3 MPa was generated by a 0.01 m projectile, which was then used to initiate the wave propagation process. Along the direction of the wave propagation ( z -direction), a series of axial middle cross-sections were selected to visualize the wave traveling process in terms of pressure and axial flow velocity distribution at different time steps (Figure 6). Here, we set $=l_{tube}/c_{f}$ , and the displayed time steps were $t_{1}=0.25\;T,\;t_{2}=0.50\;T,\;and\;t_{3}=0.75\;T$ . After the impact of the projectile, the pressure jumped to ∼ 3.2 MPa rapidly, accompanied by a relatively low level of flow velocity (less than 2.3 m/s). As shown in Figure 5, peak pressures at the different time steps did not show an apparent dissipation, while the maximum axial flow velocity slow down slightly. Details on the dissipation values are discussed in Section 3.3. The phase differences calculated by Eq. 7 showed that the wave traveled at an acoustic speed.

### Stress distribution analysis of wall/cell cultured area

The bottom wall was defined as a smooth wall area for cell culture. The results at t=0.75 T were selected to display the stress distribution in space. Figure 6A shows the compression pressure applied on the bottom wall, which appears to be similar to the compression pressure distributed in the axial plane in Figure 5A. The pressure distributed was almost identical to that in the transverse direction ( -direction) (Figure 6B). The axial shear stress ( $\tau_{z}$ ) is shown in Figure 6C, which exhibited no obvious difference in the transverse direction. The maximum axial shear stress at t=0.75 T was nearly 1.9 kPa, which was much higher than the maximum transverse shear stress ( $\left|\tau_{x,\max}\right|<1$ Pa). From these observations, it was inferred that the transverse shear stress ( $\tau_{x}$ ) could be neglected, even though it showed an apparent concentration of stress at the edges (Figure 6B).

### Effect of cells on stress spatial distribution

To evaluate the influence of cultured cells on the spatial distribution of stress on the bottom wall, the relationship between the two distributions and the density and height of the cells could be written as:

$$\begin{array}{l} \sigma =f\left(\delta_{cell},\rho_{cell}\right) \end{array}$$ (44)

**FIGURE 7:**
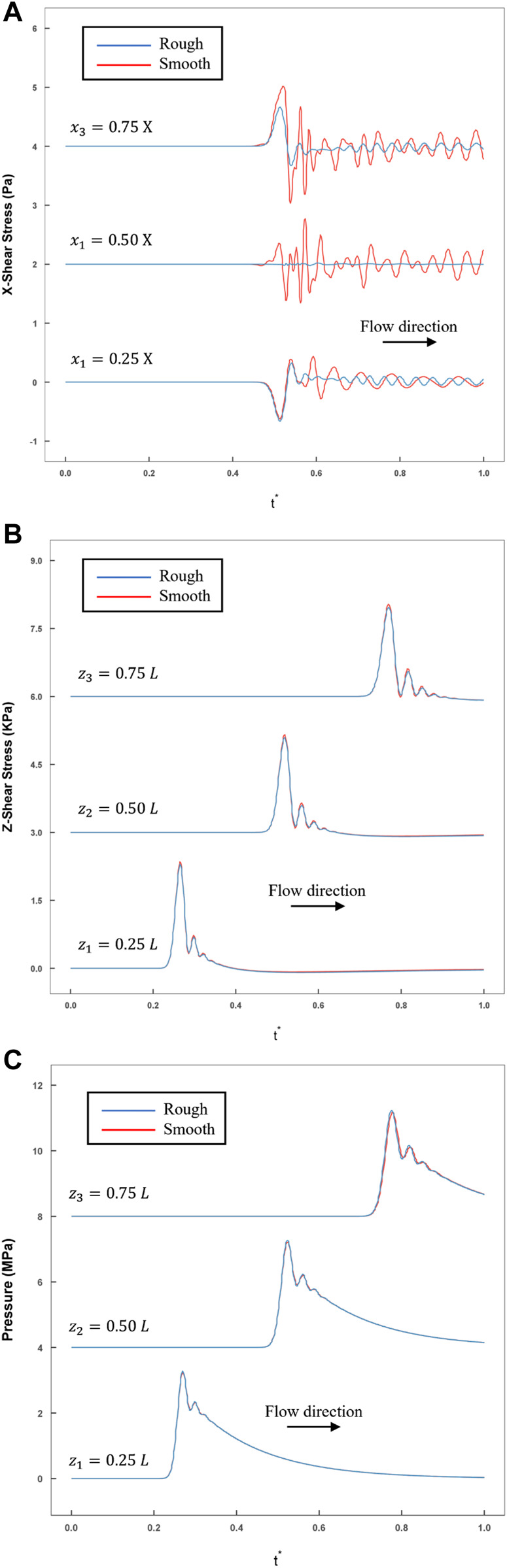
The comparison of rough and smooth model historical variations in stress were recorded by monitoring the points calculated by FLUENT (blue line: rough model; red line: smooth model). **(A)** The transverse shear stress recorded at three monitoring points ( $x_{1}=0.25\;X$ , $x_{2}=0.5\;X,$ $x_{3}=0.75\;X$ ) at the line z=0.5 L ; **(B)** The axial shear stress recorded at three monitoring points ( $z_{1}=0.25\;l$ , $z_{2}=0.5\;l$ , $z_{3}=0.75\;l$ ) at the line x=0.5 X : **(C)** Compression stress recorded at three monitoring points ( $z_{1}=0.25\;l$ , $z_{2}=0.5\;l$ , $z_{3}=0.75\;l$ ) at the line x=0.5 X .

Where σ is the compression and shear stress on the bottom wall; $\delta_{cell}$ is the average height of the cells, and $\rho_{cell}$ is the density of the cells. Normally, the height of the cells, $\delta_{cell},$ was consistent at ∼ $1\times 10^{-5}$ m and the interval of variation, $\rho_{cell},$ was [0,∞) . The two boundary conditions were defined as follows: the lower limit situation, i.e., $\rho_{cell}=0$ (no cell cultured), represented a smooth wall; the upper limit situation, i.e., $\rho_{cell}=\infty$ , denoted that countless cell had been cultured on the bottom wall and imparted a roughness of $1\times 10^{-5}$ m. By comparing the stress in these two situations, the effect of cells on stress distribution could be assessed (Figure 7). Along the axial and transverse directions, several monitoring points were set to calculate the compression and shear stress: σ (3 points along the axial direction at x=0.5 X : $z_{1}=0.25\;l$ , $z_{2}=0.5\;l$ , $z_{3}=0.75\;l$ ; 3 points along the x direction at z=0.5 L : $x_{1}=0.25\;X$ , $x_{2}=0.5\;X$ , $x_{3}=0.75\;X$ ).

In addition, the temporal variations in $\tau_{x}$ in the transverse direction were also recorded. As the maximum of $\tau_{x}$ was quite small (did not exceed $10^{1}$ Pa) within the interval of (0.25 X,0.75 X) , $\tau_{x}$ could be neglected (Figure 7A). As shown in Figures 7B,C, the compression and axial shear stress variations along the axial direction were recorded at the monitoring points, and the same changing trend was observed. Exact statistics on the maximum values are shown in Table 1. Regardless of the maximum value of pressure or axial shear, there was almost no difference between these two conditions in an interval of (0.25 l,0.75 l) . The $\rho_{\exp}$ was in the interval of $0<\rho_{\min}\leq \rho_{\exp}\leq \rho_{\max}\ll \infty$ . Therefore, the smooth boundary condition would be much closer to the actual experimental observations.

**TABLE 1:** Maximum values of the monitoring points were calculated by FUENT.

	Compression			Axial shear		
	$z_{1}$	$z_{2}$	$z_{3}$	$z_{1}$	$z_{2}$	$z_{3}$
Rough	3.28 MPa	3.26 MPa	3.23 MPa	2.29 kPa	2.09 kPa	1.97 kPa
Smooth	3.24 MPa	3.21 MPa	3.14 MPa	2.35 kPa	2.16 kPa	2.01 kPa
Average ratio (Smooth/Rough)	1.019±0.0782			0.975±0.0029		

Table 1 shows the attenuation of pressure and axial shear stress along the axial direction. In this model, the average reduction was within 1% in every 0.25 l ( 15 mm) for pressure and ∼ 6% in every 0.25 l for shear stress.

**FIGURE 8:**
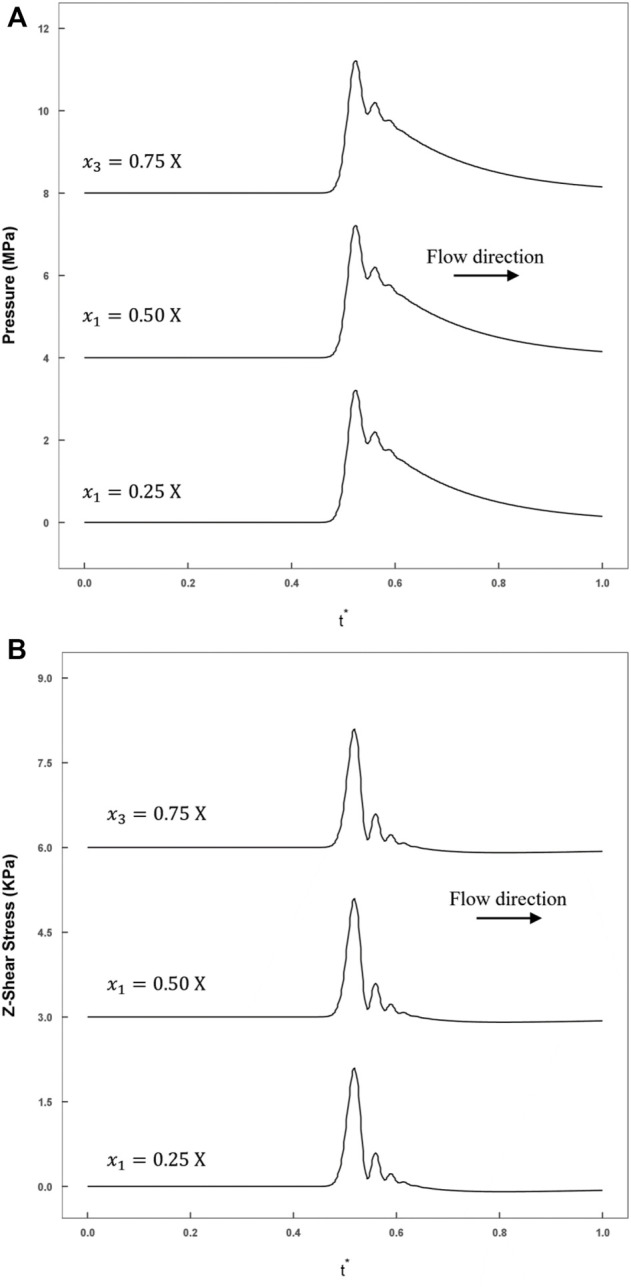
The smooth model historical variations in stress recorded by monitoring points calculated by FLUENT (blue line: rough model; red line: smooth model). **(A)** The axial shear stress recorded at three monitoring points ( $x_{1}=0.25\;X$ , $x_{2}=0.5\;X,$ $x_{3}=0.75\;X$ ) at the line z=0.5 L ; **(B)** The compression stress recordings at three monitoring points ( $x_{1}=0.25\;X$ , $x_{2}=0.5\;X,$ $x_{3}=0.75\;X$ ) at the line z=0.5 L .

In addition, the temporal variations in the transverse direction are shown in Figure 8, where the monitoring points were set in the middle cross-section ( z=0.5 l ). The attenuation of either the pressure or the axial shear stress in the transverse direction was less than 1% . These results imply the great repeatability of this model.

## Discussion

In the past, many studies have focused on revealing the mechanical properties of cells with the assistance of interdisciplinary techniques, such as solid mechanics, image processing, and fluid mechanics. Such works have implied that as compared to static or quasi-static loadings, the mechanical properties of cells are likely to show a distinct behavior difference under dynamic loading ( $\dot{\epsilon }>10^{0}\;s^{-1}$ ). Considering the length scale of cells, it is difficult to clamp a single cell using traditional dynamic loading methods, such as Split Hopkinson’s Pressure Bar (SHPB). Hence, the challenge is how to apply controllable dynamic loading on cells properly and the real-time measurement of cell response during this process.

In this paper, we have proposed a new method for the application of dynamic loading that couple’s compression and shear stress synchronously on isolated cells under normal culture conditions. This method describes a case, where an impact loading from a projectile was employed to generate a pressure wave, together with corresponding shear stress due to the fluid viscosity. At the same time, the strain variations in the cell could be captured by a high-speed camera. Eventually, data on the essential stress and strain on the cells were collected to explore the mechanical properties of cells. We established a representative model (rectangular channel filled with water) as shown in Figures 1A,B. With this model, we explained two main questions as elaborated on in Section 2 and Section 3 on the working of this method and its known limitations:

1) How can the weak shock wave generated by projectile impact be calculated? Does the solid structure (channel) affect the flow and how can it reduce the fluid-structure interaction (FSI)?
2) Does the shear stress keep the same phase as the compression stress? Does the existence of cells have a strong influence on stress distribution? How is this related to the positions along the axial and transverse directions?

The basic purpose was to explore the biomechanical mechanism of single cell response to dynamic mechanical loading, where the length scale was focused on $10^{-6}$ m and the cells were treated as a homogenous element. This length scale had to be taken into consideration due to its influence on the fluid domain meshing and CFD (actually, more factors had been considered during the meshing process). The characteristic length of the channel we designed was $3\times 10^{-3}$ m, which was much larger than the size of the cells. Generally, the pressure and wall shear stress were directly assumed when the stresses were applied to the cells, while the approximate treatment relied on the hypothesis that the flow was hydraulically smooth. The estimated average height of the cells was ∼ $1\times 10^{-5}$ m, therefore, one of the extremely idealized hypotheses was that the bottom wall was occupied by countless cells that were equivalent to a $1\times 10^{-5}$ m roughness on the bottom wall. The total pressure and axial shear stress showed that there was only a slight difference between the smooth and rough conditions (within 2% ) (Figure 7). In the actual experimental process, the cell density only sparsely facilitated the capture of the strain field by a high-speed camera. Therefore, the effect of cells on the fluid flow could be neglected.

The Doppler effect is commonly used to detect the flow velocity of a flowing fluid, but experimental techniques may render it less reliable on small velocities and the changing profile near the walls (Riasi et al., 2009). Several sophisticated numerical models have been established to more accurately explain the details of the transient flow (Ghidaoui and Kolyshkin, 2001; Martins et al., 2018). It has been proven that CFD performs very well in modeling the pressure wave traveling processes (Mandair, 2020). Therefore, we established the CFD model to help us evaluate the proposed method, and we will consider these evaluation results as a very important reference for future experimental work.

The boundary conditions should be comprehensively considered in CFD modeling as we have described in detail in Section 2 of this article. The criteria of channel size design were not too strict, which allowed us to adjust the details freely according to the actual lab environment, provided that the size details obeyed the above-mentioned limitations of the Knudson number to continuously maintain the fluid. As for the projectile, its material should be kept the same as that of the buffer. Figure 4 shows the means of controlling the amplitude and decay time constant of the initial pressure by the projectile.

The water hammer is a well-known problem. The weak shock wave generated in this problem would induce a sudden pressure jump corresponding to the low flow velocity. Accordingly, we described the stress wave propagation in total pressure and the flow velocity forms; we also visualized this process in the middle axial section (Figure 5). In this process, the dissipation effect was mainly due to the fluid viscosity and could not be neglected. We selected several points in the z and x directions on the bottom wall to figure out the proper areas for cell measuring (Figures 6–8). Only the z shear stress changed slightly ( 6% drop at every 15 mm in this example), and the x shear stress was so small that it could be neglected (in the interval of 0.25−0.75 X ). These CFD results give us a high fault tolerance rate in repeating experiments.

## Conclusions

In this paper, we focused on the application of dynamic loadings on single cells and revealed their mechanical response. Based on the Water-Hammer theories, we have developed a novel experimental method with a corresponding CFD model to help investigate cell mechanics under dynamic loadings. In this method, cells were normally cultured inside a microchannel. After impact, the stress wave generated applied a coupled compression-shear stress on an isolated living cell inside the microchannel. The results from an example model showed that the stress conditions could be easily controlled by controlling the velocity or length of the projectile. In addition, this method will allow researchers to adjust various design elements of their channels, such as the size, materials, etc., according to their lab’s environmental and actual needs, if the new design meets the relevant criteria presented in this study. This model offers repeatability, as a wide area is available for cell strain capturing, where cells suffer from nearly the same stress loadings.

## Data Availability

The original contributions presented in the study are included in the article/supplementary material, further inquiries can be directed to the corresponding authors.
